# Predictive value of a series of inflammatory markers in COPD for lung cancer diagnosis: a case-control study

**DOI:** 10.1186/s12931-019-1155-2

**Published:** 2019-08-28

**Authors:** Cecilia Mouronte-Roibás, Virginia Leiro-Fernández, Alberto Ruano-Raviña, Cristina Ramos-Hernández, Pedro Casado-Rey, Maribel Botana-Rial, Esmeralda García-Rodríguez, Alberto Fernández-Villar

**Affiliations:** 1 Pulmonary Department, Hospital Álvaro Cunqueiro, Vigo Health Area; NeumoVigoI+i Research Group, Vigo Biomedical Research Institute (IBIV), Vigo, Spain; 2 University of Santiago de Compostela, Preventive Medicine and Public Health. School of Medicine, San Francisco st s/n Santiago de Compostela, A Coruña, Spain; 3 CIBER de Epidemiología y Salud Pública, CIBERESP, Madrid, Spain; 4 Clinical Analysis Department, Hospital Álvaro Cunqueiro, Vigo Health Area, Vigo, Spain

**Keywords:** Lung cancer, COPD, Inflammation, Neutrophils, A1AT, Cholesterol

## Abstract

**Background:**

There is a relationship between Chronic Obstructive Pulmonary Disease (COPD) and the development of lung cancer (LC). The aim of this study is to analyse several blood markers and compare their concentrations in patients with only COPD and LC + COPD.

**Methods:**

Case-control study with cases presenting combined LC and COPD and two control groups (patients presenting only COPD and patients presenting only LC). We also included LC patients with descriptive purposes. In both groups, peripheral blood analyses of TNF-α, IL-6, IL-8, total leukocyte, lymphocyte and neutrophil counts, neutrophil-to-lymphocyte ratio, total platelet count, mean platelet volume, platelet-to-lymphocyte ratio, alpha 1-antitripsin (A1AT), IgE, C-reactive protein, fibrinogen, cholesterol and bilirubin were performed. We developed univariate and multivariate analyses of these markers, as well as a risk score variable, and we evaluated its performance through ROC curves.

**Results:**

We included 280 patients, 109 cases (LC + COPD), 83 controls (COPD) and 88 LC without COPD. No differences were observed in the distribution by sex, age, BMI, smoking, occupational exposure, lung function, GOLD stage or comorbidity. Patients with LC + COPD had significantly higher levels of neutrophils [OR 1.00 (95%CI 1.00–1.00), *p* = 0.03] and A1AT [OR 1.02 (95%CI 1.01–1.03), *p* = 0.003] and lower cholesterol levels [OR 0.98 (95%CI 0.97–0.99), *p* = 0.009] than COPD controls. We developed a risk score variable combining neutrophils, A1AT and cholesterol, achieving a sensitivity of 80%, a negative predictive value of 90.7% and an area under the curve of 0.78 (95%CI 0.71–0.86).

**Conclusions:**

COPD patients who also have LC have higher levels of neutrophils and A1AT and lower of cholesterol. These parameters could be potentially predicting biomarkers of LC in COPD patients.

**Electronic supplementary material:**

The online version of this article (10.1186/s12931-019-1155-2) contains supplementary material, which is available to authorized users.

## Background

Lung cancer (LC) is the leading cause of cancer death worldwide, with a 5-year survival of approximately 15%. Chronic Obstructive Pulmonary Disease (COPD) is the fourth cause of death worldwide, with a current prevalence around 10% [1–3]. LC mortality is explained by the fact that most diagnoses are made in advanced stages, being able to identify tumors in localized stages only in 16–22% of cases, although new diagnostic techniques and the implementation of rapid diagnostic units are increasing the proportion of patients diagnosed with localized LC [2, 4]. In these cases, survival can reach up to 55.6% at 5 years [5].

Some studies have demonstrated that COPD is a risk factor for LC development, independently of tobacco exposure. In addition, COPD and LC share some common features. Smoking is the main cause of both diseases, COPD affecting 15–20% of smokers, while 80% of LC patients are smokers or ex-smokers. Besides tobacco use, COPD and LC share some genetic backgrounds, environmental exposures, and common underlying inflammatory processes [6, 7].

Airway chronic inflammation is one of the pathophysiological mechanisms that plays a key role in the amplification of the initial mutagenic response of LC. It is possible that persistent airway inflammation in COPD patients induces alterations in the bronchial epithelium which favor carcinogenesis [8]. In patients with COPD and in smokers, the expression of certain cytokines is increased, such as IL-6 and IL-8, which, in turn, through the induction of the enzyme cyclooxygenase-2, promote an inflammatory response in lymphocytes. They can also inhibit apoptosis, interfere with cellular repair mechanisms and promote angiogenesis, contributing to neoproliferative processes [8]. Other cytokines and growth factors such as tumor necrosis factor (TNF)-α have also been shown to participate in the development, tumor growth and metastasis of LC in patients with underlying respiratory conditions [9]. In fact, various blood markers of inflammation have been evaluated separately in patients either with COPD or with LC and other malignant tumors (Additional file 1: Table S1). These markers include C-reactive protein (CRP), platelet, neutrophil, and lymphocyte numbers but especially include neutrophil/lymphocyte ratio (NLR), platelet/lymphocyte ratio (PLR), mean platelet volume (MPV), alpha-1-antitripsin (A1AT), fibrinogen, cholesterol or bilirubin [10–14]. Therefore, the increased risk of developing LC in patients with COPD could be related to the existence of a previous inflammation, making them more susceptible to the carcinogenic components of tobacco. This inflammation persists even years after having stopped smoking, which may be a cause of LC in ex-smokers [8]. To our knowledge, there are no studies assessing a complete series of inflammatory blood markers in patients with COPD comparing them with LC + COPD patients. To have some predictive markers in COPD patients showing a higher possibility of LC would mean an early diagnosis and therefore improving their clinical results. We have selected 16 biomarkers in order to test the importance of persistent airway inflammation in the development of LC in COPD patients, as these markers have already shown to be high in COPD and related to disease progression, prognosis and response to treatment in LC.

The aim of this study is: 1) to assess a panel of different markers (IL-6, IL-8, TNF-α, CRP, PCR, IgE, platelet, neutrophil, and lymphocyte numbers, NLR, PLR, MPV, A1AT, fibrinogen, cholesterol and bilirubin) in three groups of patients (COPD, patients with COPD and LC [LC + COPD] and LC without COPD), focusing on the comparison between COPD and LC + COPD patients and, 2) to select those markers associated with LC + COPD and to create a score to predict the risk of presenting LC based on selected clinical parameters.

## Methods

### Study design and case and control selection

This is a case-control study in which patients with COPD, with LC + COPD and with LC only were included from September 2014 to May 2018 from the Vigo University Hospital. This hospital attends a 450,000-inhabitant area, and the pulmonary department applies practically all pulmonary techniques and procedures. Cases were COPD patients with synchronic LC (LC + COPD) diagnosed in the Lung Cancer Rapid Diagnosis Unit (LCRDU), while controls (patients with COPD and no evidence of LC) were captured in a general pulmonary consultation that was carried out on the same days as the LCRDU, including patients with recently-diagnosed COPD (less than 6 months). We included a second group of controls with LC with normal lung function to make a descriptive comparison of inflammatory marker’s levels between the three groups. The LCRDU permits a diagnostic and staging process of LC and other thoracic neoplasms. This unit assesses around 95% of all LC patients in our area.

Patients with symptoms or evidence by imaging tests of active infection, ischemic or congestive heart disease, thromboembolic disease or other underlying inflammatory processes (outbreak of connective tissue disease, inflammatory bowel disease...), as well as patients with a second synchronous tumor were excluded from the study to avoid false positives when assessing blood markers. In addition, we also excluded all patients with advanced or very symptomatic tumor disease requiring hospital admission (hepatic failure, moderate or massive hemoptysis, superior vena cava syndrome or metastatic disease requiring urgent treatment, such as palliative radiotherapy for bone or brain metastases). Therefore, all patients included in the study were managed on an outpatient basis in the LCRDU. Also, we excluded patients presenting with a microbiological isolation in any of the samples carried out during the process (sputum cultures or cultures of bronchoscopy or surgery samples). However, no cut-off points were established as exclusion criteria in the levels of the biomarkers studied, in order to avoid intervening in the results of the study.

The diagnosis of LC was made after suggestive radiological findings with pathologic confirmation [15]. COPD was defined following the *Global Initiative for Obstructive Lung Disease* (GOLD) recommendations as the presence of persistent respiratory symptoms and a forced expiratory volume in the first second (FEV_1)_/ forced vital capacity (FVC) ratio < 0.70 after a bronchodilator test [16]. We excluded patients unwilling to participate or to donate blood samples, those with contraindications or incapable of performing spirometric tests correctly, and patients with any other pulmonary obstructive disease other than COPD.

We assessed a panel of different blood markers in the three groups of patients: IL-6, IL-8, TNF-α, CRP, IgE, platelet, neutrophil, and lymphocyte numbers, NLR, PLR, MPV, A1AT, fibrinogen, cholesterol and bilirubin, and whether patients were receiving growth factors or statins. All biomarkers were tested in a stable phase, in outpatients, without any concomitant infection or inflammatory process, and without any synchronous tumor.

### Information retrieval

Collected data included basic demographics: age, gender, tobacco history, functional variables (comorbidity assessed by the Charlson Comorbidity Index [17], FEV_1_, DLCO and body mass index (BMI). The histological type of LC was also included by reviewing the pathology report, as well as the stage at diagnosis according to the TNM eight edition’s descriptors [15] after complete staging processes. Other data necessary for COPD characterization were included, such as the GOLD and *Spanish Guideline for COPD* (GesEPOC) classifications valid at the study onset [16, 18], COPD assessment test (CAT) [19] and BODEx index [20].

Smokers were defined as participants who had smoked 100 or more cigarettes in their lifetime. Current smokers were those who smoked more than one cigarette in the month prior to enrollment or quit within one year of enrollment. The remaining smokers were classified as ex-smokers. Never smokers were defined as having smoked less than 100 cigarettes in their lifetime [21].

Spirometry was performed at the time of inclusion in the study by a technician specialized in respiratory functional tests. It was carried out with a Masterlab pneumatic-type spirometer (Jaeger AG, Wuezburg, Germany), using acceptability and reproducibility criteria from SEPAR and ERS [22] guidelines, with Quanjer Gli reference values ( [23]). A bronchodilator test was performed in all cases, by administrating 400 μg of salbutamol in 4 puffs (100 μg per puff) at 30 s intervals.

Emphysema was determined through computed tomography (CT) assessment by experimented radiologists. The CT studies were performed in two devices: Lightspeed VCT of 64 rows of detectors (GE Medical Systems, Milwaukee, Wisconsin) and Somatom Emotion of 16 rows of detectors (Siemens Medical Solutions, Enlargen, Germany).

Peripheral venous blood was collected from all patients into Vacutainer tubes in the morning. Serum was obtained by centrifugation of whole blood at 3000 g for 10 min. Plasma (CITRATE as anticoagulant) was obtained by centrifugation at 3500 g for 15 min at a temperature of 4 °C. Serum samples used to measure IL-6, IL-8 and TNF-α levels were stored at − 80 °C until they were analyzed. IL-6, IL-8 and TNF-α serum concentrations were determined by validated immunoassays (IMMULITE ONE, Siemens, Germany), full blood counts were carried out using ADVIA 2120 (Siemens, Germany); serum CRP, cholesterol and bilirubin were measured using ADVIA 2400 (Siemens, Germany); A1AT was analyzed by nephelometric assay (IMMAGE, Beckman Coulter, USA); IgE levels were measured by fluorometric immunoassay (PHADIA 250, Thermo Scientific, USA) and fibrinogen was calculated in ACL TOP 700 instrument (Werfen Company, Spain) Limits of detection (LOD) for IL-6, IL-8 and TNF-α were 2 pg/ml, 5 pg/ml and 4 pg/ml. Biomarker concentrations were below the LOD in some individuals. To avoid a downward bias of the population data, a nominal level of half of the LOD value was used in the analysis in individuals with values below the LOD [24].

### Statistical analysis

The design and statistics of the study were reviewed by a professor of epidemiology who is a co-author of the manuscript, and who has an extensive experience in case-control studies. We first carried out a descriptive analysis of levels of all markers in the three groups of patients through the use of boxplots. Then we developed a univariate analysis to evaluate differences between cases (patients with LC + COPD) and controls (COPD) for all assessed variables. The t-student test was used for quantitative variables and Chi^2^ test was used to compare percentages for qualitative variables. Our limit of significance was *p* < 0.05. We included variables with a *p* < 0.10 in the multivariate models (performed through a forward conditional method), developing interaction analyses for all of them. For the final significant variables, we performed two multivariate logistic regression models, the first adjusting for age and sex and the second also including the remaining variables. To do so, the significant quantitative variables were stratified into terciles for inclusion in the logistic regression models. Application of this multivariate analyses led to the design of a risk score variable for each given patient based on the results of the multivariate logistic regression. Points for a given patient were obtained by summing all the points for each predictor variable, adjusted for sex and age. Then we developed a ROC curve and assessed sensitivity, specificity and predictive values for the risk score variable, taking into account a prevalence of LC in patients with COPD of 25% [7]. The analysis was performed with SPSS 21.0 (IBM Corporation, Armonk, New York).

## Results

We included 280 patients: 109 cases (LC + COPD), 83 controls (COPD) and 88 LC patients. A descriptive and univariate analysis comparing baseline characteristics and marker levels of cases and controls is included in Table 1. As shown in the table, baseline characteristics of both groups were very homogeneous, with no relevant differences between groups, also in terms of baseline treatments. There were no patients undertaking any growth factor. One case and two controls had A1AT < 90 mg/dl. Five patients had cachexia, four were cases (one in the group with high cholesterol levels and three with medium cholesterol levels) and one was a control. Baseline characteristics of LC patients without COPD are included in Table 2.

**Table 1:** Univariate analysis comparing characteristics of cases and controls

	Cases (LC + COPD)	Controls (COPD)	*p*
Baseline characteristics			
Gender (male), *n* (%)	95 (87.1)	65 (78.3)	0.07
Age, mean (SD)	67 (10.3)	64.6 (9.4)	0.10
BMI, mean (SD)	26.6 (4.2)	27.3 (4.5)	0.29
Laboral exposure, n (%)	43 (39.4)	25 (30.1)	0.31
Tobacco history, n (%)	109 (100)	82 (98.8)	0.43
Active smokers, n (%)	62 (56.9)	45 (54.2)	0.39
Pack-years, mean (SD)	49.5 (23.8)	47.5 (25)	0.61
Emphysema, n (%)	68 (62.4)	38 (45.8)	0.32
GOLD I-II, n (%)	87 (79.8)	66 (79.5)	0.55
GesEPOC A (%)	105 (96.3)	80 (96.4)	0.65
Bodex, mean (SD)	1 (1.5)	0.8 (1.2)	0.30
CAT, mean (SD)	10.6 (6.2)	9.7 (7.6)	0.69
FEV_1_ (%), mean (SD)	69.1 (21.1)	71 (20.2)	0.55
DLCO (%), mean (SD)	68.1 (21)	70.4 (22.6)	0.58
Charlson index, mean (SD)	1.0 (1.5)	0.7 (1)	0.11
Statin consumption, n (%)	40 (36.7)	31 (37.3)	0.52
Inflammatory markers			
TNF-α (pg/ml), mean (SD)	14.7 (46.4)	9.3 (7.4)	0.25
IL-6 (pg/ml), mean (SD)	10.7 (16.6)	6.2 (12.1)	0.05
IL-8 (pg/ml), mean (SD)	29.6 (44.1)	19.2 (28.8)	0.07
Leukocytes (per μl), mean (SD)	10,004,4 (11,096.3)	7706.1 (2334)	0.04
Lymphocytes (per μL), mean (SD)	2334 (1987.4)	2398.5 (965.7)	0.77
Neutrophils (per μl), mean (SD)	5920 (2469.1)	4464.2 (2136.7)	< 0.001
NLR, mean (SD)	3.1 (1.8)	2.1 (1.5)	< 0.001
Platelets (per μl), mean (SD)	295,114,1 (124,102.4)	243,402.4 (72,978.9)	0.001
MPV (fl), mean (SD)	8.4 (1.2)	8.9 (1)	0.003
PLR, mean (SD)	154 (86.6)	118.5 (72.1)	0.003
Fibrinogen (mg/dl), mean (SD)	461 (198.7)	392.5 (166.5)	0.07
A1AT (mg/dl), mean (SD)	174 (49.9)	136.8 (29.1)	< 0.001
IgE (kU/l), mean (SD)	155,7 (109.7)	177.8 (569.3)	0.78
CRP (mg/l), mean (SD)	22 (31.7)	6.1 (8.8)	< 0.001
Cholesterol (mg/dl), mean (SD)	178.3 (37.1)	201.8 (37.1)	< 0.001
Bilirrubin (mg/dl), mean (SD)	0.6 (0.3)	0.6 (0.2)	0.50

LC: lung cancer; COPD: chronic obstructive pulmonary disease; BMI: Body mass index; GOLD: Global Initiative for Obstructive Lung Disease; GesEPOC A: non exacerbators, according to the Spanish Guidelines of COPD; CAT: COPD Assessment Test; FEV1: forced expiratory volume in the first second; DLCO: carbon monoxide diffusion capacity; IL: interleukin; NLR: neutrophil/lymphocyte ratio; MPV: mean platelet volume; PLR: platelet/lymphocyte ratio; A1AT: alpha 1-antitripsin; IgE: E immunoglobulin, CRP: C reactive protein

**Table 2:** Characteristics of patients with LC only

	LC patients without COPD
Gender (male), n (%)	63 (71.6)
Age, mean (SD)	65.7 (10.9)
BMI, mean (SD)	29.3 (5.3)
Laboral exposure, n (%)	28 (33.7)
Tobacco history, n (%)	73 (83)
Active smokers, n (%)	39 (44.3)
Pack-years, mean (SD)	40.3 (23.11)
SCLC, n (%)	4 (4.9)
Adenocarcinoma n(%)	42 (54.5)
Squamous, n (%)	20 (25)
Advanced stage at diagnosis, n (%)	41 (48.2)
FEV_1_ (%), mean (SD)	87 (20.8)
DLCO (%), mean (SD)	82.2 (20.3)
Charlson index, mean (SD)	2 (2.2)

LC: lung cancer; COPD: chronic obstructive pulmonary disease; BMI: Body mass index; SCLC: small cell lung cancer; FEV_1_: forced expiratory volume in the first second; DLCO: carbon monoxide diffusion capacity

**Fig. 1:**
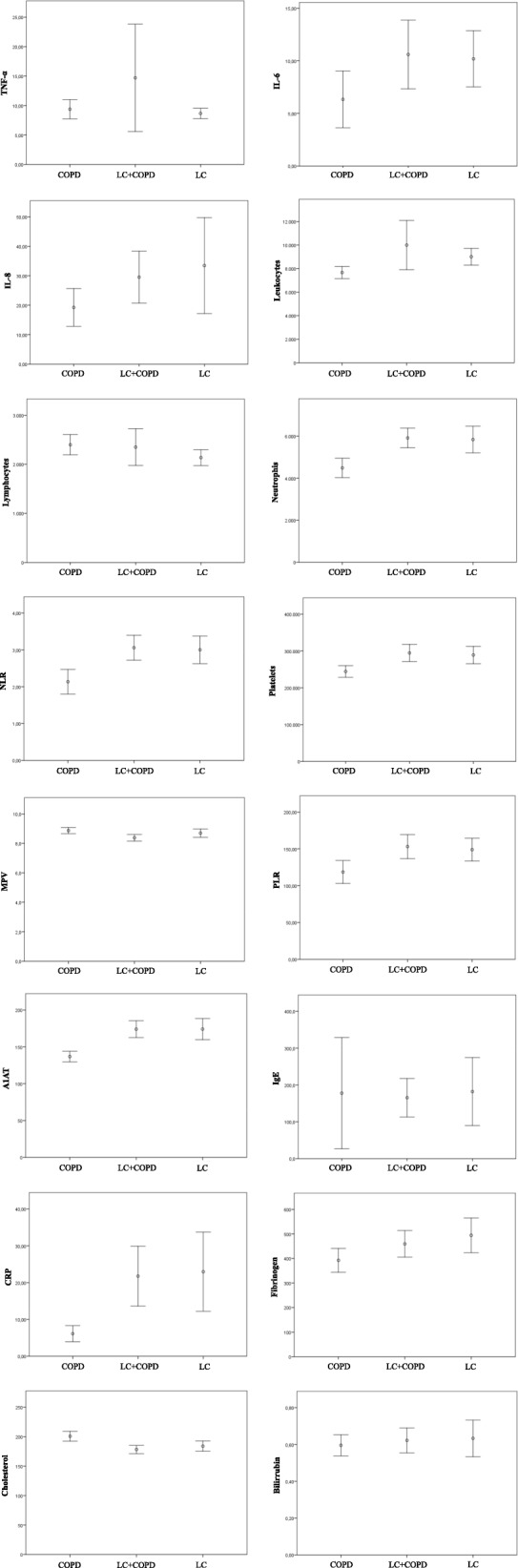
Descriptive comparison between levels of biomarkers in the three groups of patients

The most frequent histological type was adenocarcinoma, in 84 cases (44.9%), followed by squamous (26.7%), undifferentiated (18.2%), small-cell LC (SCLC) (7%) and carcinoid (3.2%). Regarding tumour characteristics, when comparing LC + COPD with LC patients, we found that patients with LC without COPD had more adenocarcinomas (54.5% vs 38.4%; *p* = 0.02), whereas patients with LC + COPD had more SCLC (17.8% vs 4.9%; *p* = 0.006). There were no differences in stage at diagnosis. Figure 1 shows descriptive boxplots for all inflammatory markers in the three groups of patients.

**Fig. 2:**
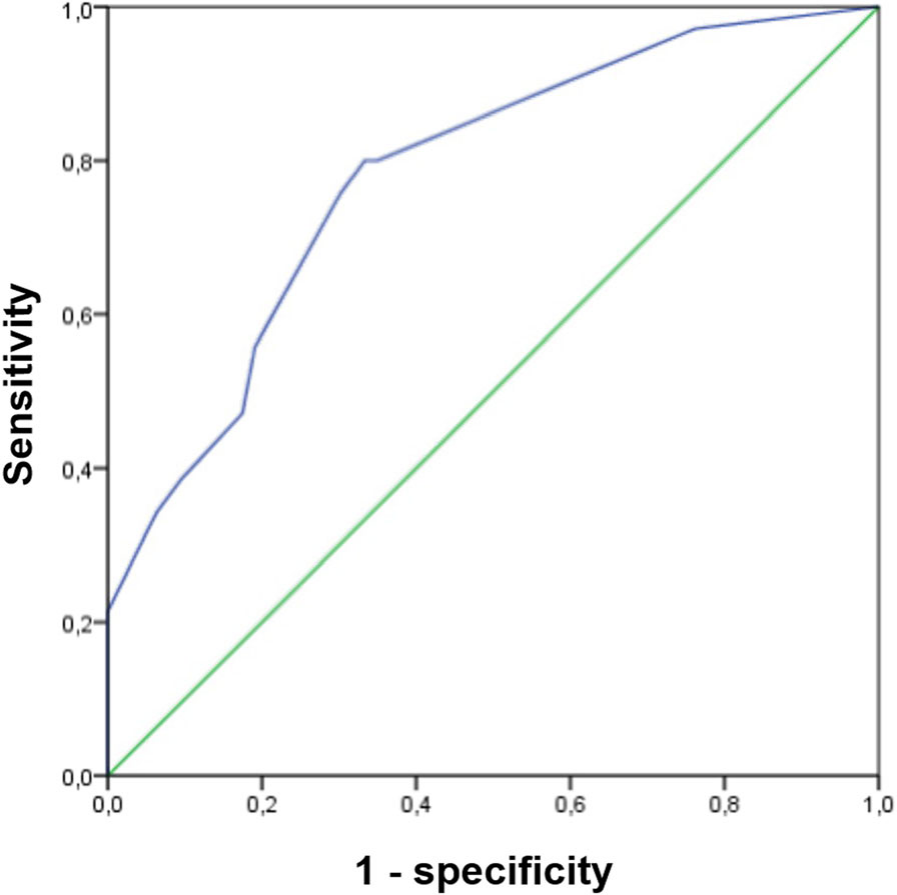
ROC curve of the risk score

We developed a multivariate logistic regression model comparing cases and controls (Table 3). High neutrophil and A1AT levels and low cholesterol levels were the only significant variables in the multivariate analysis. Therefore, those were the variables chosen for their stratification in terciles and their inclusion in the score. It can be observed that three variables are associated significantly with the probability of being a case: patients with LC + COPD had significantly higher levels of neutrophils [OR 4.90 (95%CI 1.60–14.94 for those in the highest tercile of neutrophils, *p* = 0.005] and A1AT [OR 3.6 (95%CI 1.23–10.53), for those in the highest tercile of A1AT, *p* = 0.019] and lower cholesterol levels [OR 2.91 (95%CI 1.08–7.85), for those in the lowest tercile of cholesterol, *p* = 0.03] than COPD controls. The point scoring system shown in Table 4 was used to measure the magnitude of the association of each of the significant factors in the multivariate analysis with the odds of being a case, thus leading to the development of a risk score. Performance and ROC curves of this risk score are presented on Fig. 2 and Table 5. Based on our model and assuming a prevalence of 25% among COPD patients, we reached a sensitivity of 80%, with an optimal negative predictive value (NPV) of 90.7% [7].

**Table 3:** Multivariate analysis comparing characteristics of cases and controls with variables stratified by terciles

Variable	Cases, *n* (%)	Controls, *n* (%)	OR (95%CI) ^a^	p	OR (95%CI) ^b^	p
A1AT (mg/dl)						
Low: < 138	27 (24.7)	41 (49.4)	1 (−)		1 (−)	
Medium: ≥138 and < 167	32 (29.3)	32 (38.5)	1.49 (0.66–3.39)	0.34	0.96 (0.38–2.45)	0.94
High: ≥167	50 (45.9)	10 (12)	7.33 (2.80–19.24)	< 0.001	3.60 (1.23–10.53)	0.019
Cholesterol (mg/dl)						
Low: < 168	40 (36.7)	18 (21.7)	2.91 (1.43–5.94)	0.003	2.91 (1.08–7.85)	0.03
Medium: ≥168 and < 200	44 (40.4)	25 (30.1)	3.73 (1.74–8.00)	0.001	3.03 (1.09–8.41)	0.03
High: ≥200	25 (22.9)	40 (48.2)	1 (−)		1 (−)	
Neutrophils (per μl)						
Low: < 4007	22 (20.2)	45 (54.2)	1(−)		1 (−)	
Medium: ≥4007 and < 5955	42 (38.5)	25 (30.1)	3.35 (1.65–6.84)	0.001	2.95 (1.14–7.60)	0.02
High: ≥5955	45 (41.3)	13 (15.7)	7.08 (3.18–15.77)	< 0.001	4.90 (1.60–14.94)	0.005

OR: odds-ratio; 95%CI: confidence interval of a 95%; A1AT: alpha 1-antitripsin; ^a^ OR adjusted by age and gender; ^b^ OR adjusted by age, gender, alpha 1-antitripsin, cholesterol and neutrophils

**Table 4:** Point scoring system for predicting the risk of being a case

Characteristic	Points assigned*
High A1AT levels (≥167 mg/dl)	4
Low and medium cholesterol levels (< 200 mg/dl)	3
Medium neutrophil levels (≥4007 and < 5955 per μl)	3
High neutrophil levels (≥5955 per μl)	5

A1AT: alpha-1 antitripsin

*A total point score for a given patient is obtained by summing all the points for each applicable characteristic. The points assigned to each predictor variable were based on coefficients obtained from the logistic-regression model adjusted for age, sex, alpha 1-antitripsin, cholesterol and neutrophils exposed in Table 3

**Table 5:** Sensitivity, specificity and predictive values of the risk score

	AUC (95%CI)	Cut-off value	S (%)	Sp (%)	PPV (%)	NPV (%)
Risk score*	0.78 (0.71–0.86)	> 3.5 points	80	65.1	43.5	90.7

* The points assigned to each predictor variable were based on coefficients obtained from the logistic-regression model adjusted for age, sex, alpha 1-antitripsin, cholesterol and neutrophils; S: sensitivity; Sp: specificity; PPV: positive predictive value; NPV: negative predictive value, AUC: area under the curve; CI: confidence interval

We repeated the univariate and multivariate analyses excluding patients with advanced LC stage, to minimize the effect of higher inflammation levels in this kind of tumours. We found that in local LC + COPD, A1AT was significantly higher than in COPD patients: OR 1.02 (1.00–1.03); *p* = 0.03, with an AUC of 66.4 (Table 6).

**Table 6:** Univariate and multivariate analyses comparing characteristics of cases and controls with local LC

UNIVARIATE ANALYSIS			
	Cases (localized LC + COPD)	Controls (COPD)	p
Baseline characteristics			
Gender (male), n (%)	31 (83.7)	65 (78.3)	0.330
Age, mean (SD)	65.5 (12.9)	64.6 (9.4)	0.680
BMI, mean (SD)	27.7 (4.3)	27.3 (4.5)	0.510
Laboral exposure, n (%)	15 (40.5)	25 (30.1)	0.390
Tobacco history, n (%)	37 (100)	82 (98.8)	0.690
Active smokers, n (%)	20 (54.1)	45 (54.2)	0.340
Pack-years, mean (SD)	45.9 (24)	47.5 (25)	0.750
GOLD I-II, n (%)	5 (13.5)	66 (79.5)	0.260
GesEPOC A (%)	35 (94.6)	80 (96.4)	0.490
Bodex, mean (SD)	0.9 (1.50)	0.8 (1.2)	0.700
CAT, mean (SD)	8.5 (3)	9.7 (7.6)	0.570
FEV_1_(%), mean (SD)	71.6 (21.3)	71 (20.2)	0.880
DLCO(%),mean (SD)	72.6 (22)	70.4 (22.6)	0.660
Charlson index, mean (SD)	1.3 (1.9)	0,7 (1)	0.010
Inflammatory markers			
TNF-α (pg/ml), mean (SD)	11.6 (21)	9.3 (7.4)	0.530
IL-6 (pg/ml), mean (SD)	6.5 (6.1)	6.2 (12.1)	0.850
IL-8 (pg/ml), mean (SD)	22.1 (46.4)	19.2 (28.8)	0.740
Leukocytes (per μl), mean (SD)	8880.9 (4039.1)	7706.1 (2334)	0.110
Lymphocytes (per μL),mean (SD)	2744.9 (3190.1)	2398.5 (965.7)	0.370
Neutrophils (per μl), mean (SD)	5491.1 (2324.7)	4464.2 (2136.7)	0.020
NLR, mean (SD)	2.9 (1.9)	2.1 (1.5)	0.030
Platelets (per μl), mean (SD)	273,594.6 (132,871.4)	243,402.4 (72,978.9)	0.270
MPV (fl), mean (SD)	8.4 (1.3)	8.9 (1)	0.040
PLR, mean (SD)	135 (76.3)	118.5 (72.1)	0.260
Fibrinogen (mg/dl), mean (SD)	395.3 (185.2)	392.5 (166.5)	0.950
A1AT (mg/dl), mean (SD)	157.9 (42.6)	136.8 (29.1)	0.030
IgE (kU/l), mean (SD)	176.5 (216.5)	177.8 (569.3)	0.990
CRP (mg/l), mean (SD)	14.7 (20.6)	6.1 (8.8)	0.009
Cholesterol (mg/dl), mean (SD)	180.1 (44.6)	201.8 (37.1)	0.010
Bilirrubin (mg/dl), mean (SD)	0.73 (0.50)	0.6 (0.2)	0.060
MULTIVARIATE ANALYSIS			
	OR	95%CI	p
A1AT (mg/dl)	1.02	1.00–1.03	0.03

LC: lung cancer; COPD: chronic obstructive pulmonary disease; BMI: Body mass index; GOLD: Global Initiative for Obstructive Lung Disease; GesEPOC A: non exacerbators, according to the Spanish Guidelines of COPD; CAT: COPD Assessment Test; FEV1: forced expiratory volume in the first second; DLCO: carbon monoxide diffusion capacity; IL: interleukin; NLR: neutrophil/lymphocyte ratio; MPV: mean platelet volume; PLR: platelet/lymphocyte ratio; A1AT: alpha 1-antitripsin; IgE: E immunoglobulin, CRP: C reactive protein

## Discussion

Our results suggest that a panel of 3 biomarkers out of a panel of 16, which are easy to assess, might be able to detect LC in patients presenting COPD. As we have previously stated, evidence suggests that COPD is a risk factor for developing LC [7], and one of the underlying mechanisms described is inflammation. Chronic inflammation has long been associated with carcinogenesis, contributing to 25% of all human cancers [8]. We have observed that neutrophils, A1AT and cholesterol are associated with the risk of LC in COPD patients, and if they are used combined they might predict LC risk with an AUC close to 80%. If confirmed in other studies, these results could be relevant since LC is frequent among COPD patients and their use might detect the disease in earlier stages predicting a better clinical outcome.

Evidence suggests that COPD is a risk factor for developing LC [7], and one of the underlying mechanisms described is inflammation. Chronic inflammation has long been associated with carcinogenesis, contributing to 25% of all human cancers [8] and systemic inflammation has also been shown to be a relevant manifestation of COPD [25].

As exposed in Additional file 1: Table S1, several markers have shown associations with both COPD and LC. Leukocytes, TNF-α, IL-6, IL-8, cholesterol, bilirubin and fibrinogen levels increase mortality in COPD patients, whereas white blood and platelet markers are associated with a risk of COPD exacerbations [10, 11, 26–28]. Also, elevated IgE levels can be found in COPD patients [29]. In addition, TNF-α, IL-6, IL-8, NLR, PLR, IgE have been associated with LC risk in healthy subjects, being IL-6, lymphocytes, neutrophils, NLR, platelets, PLR, fibrinogen, A1AT, CRP and bilirubin poor prognostic factors in LC patients [11, 14, 30–32]. According to our results (Fig. 1), we found differences in marker levels in patients with LC + COPD, and even in LC patients without COPD, such as IL- 6, leukocytes, PLR, fibrinogen, neutrophils, NLR, platelets, A1AT and CRP. This may indicate that these markers seem to be more related to the existence of LC than to COPD itself.

In the multivariate analysis we found that some markers were statistically associated with LC onset: higher levels of neutrophils and A1AT and lower cholesterol levels.

Lymphocytes play a crucial role in the cell-mediated host immune response to tumors. Infiltration of tumors by lymphocytes correlates with better prognosis in some cancers, although disease progression is associated with high leukocyte and neutrophil count [11]. Neutrophils support angiogenesis by secreting proangiogenic factors or proteolytic activation of such factors. Also, they ensure the collection of epidermal growth factor (EGFR), transforming growth factor-β1 (TGF- β1), platelet-derived growth factors that contribute to tumorigenesis. Neutrophils contain both pro- and anti-tumor subpopulations [11]. Neutrophil counts are known to be an independent indicator of poor prognosis in LC patients, whereas low neutrophil counts are associated with longer survival [33].

Most of the literature available on the relationship between A1AT and COPD or LC focuses on the deficit of this protein [34]. However, its role as an inflammatory marker when it presents high levels has been less studied. Possible carcinogenic mechanisms have been suggested from the excess activity of neutrophil elastase [34], which induces tissue damage at the pulmonary level due to a protease-antiprotease imbalance. More studies are needed to establish if there is an association between the A1AT and LC risk. Nevertheless, there is evidence that A1AT promotes lung adenocarcinoma metastasis [13].

Although hyperlipidemia is a negative prognostic factor in patients with stomach and prostate cancers, very few studies have explored the significance of this in LC. In one trial, HDL, LDL and total cholesterol levels were lower in LC patients when compared with healthy controls, although only HDL levels were prognostically significant [11]. The observed results of cholesterol levels in this study has not shown to be related to statin consumption or to the presence of cachexia.

In this study, we provide a risk score for COPD patients with higher risk of CP, achieving high sensitivity and NPV. In fact, the area under curve is close to 80%, and therefore only 20% of patients using this score would be misclassified. Our approach involves the measurement of A1AT, neutrophils and cholesterol to generate a classification score for each individual to predict LC. Although we did reach high sensitivity and NPV, specificity and positive predictive value were modest. This was expected since the alteration of any of the selected markers is not specific for LC, given that they are markers that show high heterogeneity among patients, that they can be modified by the different comorbidities, and that there is an important variability that it is shown in the size of some of the confidence intervals [35].

We repeated the analyses in patients with COPD and localized LC, in order to minimize biases due to higher inflammation levels in patients with advanced LC, given that patients with local LC would be the objective in the case of an eventual LC screening. In this case, the only parameter which was significantly higher in patients with LC + COPD was A1AT, although the number of patients in the group of cases was considerably reduced (37 patients), which limits the conclusions that can be drawn from this subgroup of patients. It is therefore pending if this panel results are maintained when using LC patients at an early stage presenting COPD.

The use of risk prediction models may inform selection of subjects most likely to benefit from computed tomography screening; and risk markers such as A1AT, neutrophils or cholesterol may provide useful risk information in addition to questionnaire information on tobacco exposure history. Inflammation markers are unlikely to provide enough added risk information on their own, but in combination with other risk markers they may be useful for risk stratification.

Regarding LC characteristics, the most frequent histological type was adenocarcinoma, which goes in line with other studies [5]. SCLC was significantly higher in LC + COPD patients and there was a trend, although not significant for squamous LC in this group of patients. This study found that smoking had a significantly higher effect on the SCLC risk of COPD subjects, compared with non-COPD subjects [36]. Also, COPD status was independently associated with SCLC risk when adjusted for age, gender, and smoking. Squamous LC was also more frequent in smokers and has been associated with the presence of emphysema [37].

Our study shows several limitations, inherent to its case-control design. The number of controls (COPD) is slightly lower than the number of cases (LC + COPD), although we have a second control group of patients with LC without COPD. Furthermore, the score created should be classified as exploratory, though it has relatively high discrimination power. It has to be validated against other cohorts of patients from other settings. On the positive side, study groups are very similar regarding gender and age distribution. In addition, there are limitations derived from the nature of the markers that, as previously discussed, are not very specific and may present a great inter and intraindividual variability.

Our study also presents a series of advantages. This is the first work analyzing a panel of 16 blood markers in a subgroup of patients with underlying COPD, with adjustments by stage, histological type, emphysema and smoking. Also, we added a second control group of patients with LC, to show that some markers are more related to the existence of LC than to COPD itself. Most patients presented with a mild COPD. This is useful, as they represent the group most likely to benefit from more aggressive approaches of a malignancy. On the other hand, a very complete collection of variables was made, and the sample size is very acceptable. The whole Galician population has public health coverage, meaning there was no selection bias for our sample as we recruited more than 95% of all LC cases diagnosed in the referral area during the study period. We must consider that, despite a reasonable but modest sample size, we have included in the logistic regression model five variables of which three remained statistically significant, regardless of sex and age, which is why they are good predictors of the risk of being a patient with LC and COPD, which makes our results relevant. In addition, our risk score comprises only 3 parameters which may be analyzed routinely. This makes our risk score simple and affordable, and it may prove useful at guiding decision-making in clinical practice, such as whether to implement a LC screening system for at-risk patients, or even modify the probability of malignancy scales when evaluating a pulmonary nodule.

## Conclusions

Patients with COPD who also suffer from LC have higher levels of A1AT and neutrophils and lower cholesterol. These markers seem to be more related to the presence of LC than to COPD itself, since they are increased in patients with LC without COPD. In patients with LC + COPD at localized stage, A1AT is significantly higher. The combination of A1AT, neutrophils and cholesterol in the risk score variable presents a high sensitivity and NPV, so it can be a useful tool when identifying patients with LC + COPD. However, although sensitive, these markers are not specific of LC, and more studies are needed to inform selection of COPD subjects most likely to benefit from computed tomography screening or selection of nodules at higher risk of being malignant, as risk markers such as A1AT, neutrophils and cholesterol may provide useful risk information in addition to clinical questionnaires.

## Additional file

Additional file 1: **Table S1.** Markers selected for the development of a diagnostic panel for LC + COPD [38-43]. (DOCX 35 kb)

## Abbreviations

A1AT: Alpha-1 antitripsin
AUC: Area under the curve
CAT: COPD assessment test
COPD: Chronic obstructive pulmonary disease
CRP: C Reactive protein
CT: Computed tomography
DLCO: Carbon monoxide diffusion capacity
FEV1: Forced expiratory volume in the first second
FVC: Forced vital capacity
GesEPOC: Guía Española de la EPOC
GOLD: Global strategy for the diagnosis, management, and prevention of chronic obstructive pulmonary disease
Ig: Immunoglobulin
IL: Interleukin
LC: Lung cancer
LCRDU: Lung cancer rapid diagnosis unit
LOD: Limit of detection
MPV: Mean platelet volume
NLR: Neutrophil/lymphocyte ratio
NPV: Negative predictive value
OR: Odds ratio
PLR: Platelet/lymphocyte ratio
SCLC: Small cell lung cancer
TGF-β1: Beta-one transforming growth factor
TNF: Tumor necrosis factor
