# Supplementary material for: Predictive value of a series of inflammatory markers in COPD for lung cancer diagnosis: a case-control study
Source: Respir Res. 2019 Aug 28;20:198. doi: 10.1186/s12931-019-1155-2 (PMC6712782; doi:10.1186/s12931-019-1155-2)
Supplement: Supplementary file 1 — Table S1. Markers selected for the development of a diagnostic panel for LC + COPD [38-43]. (DOCX 35 kb) [file 12931_2019_1155_MOESM1_ESM.docx]

**SUPPLEMENTARY MATERIAL**

**Supplementary table 1. Markers selected for the development of a diagnostic panel for LC+COPD.**

| **Marker** | **Role in COPD** | **Role in LC** |
| --- | --- | --- |
| TNF-α | Mortality was 6-times higher in patients with high WBC, CRP, IL-6, IL-8, TNF- α and fibrinogen (10). | TNF- α levels are increased in patients with LC+COPD when compared to COPD and healthy patients (38). |
| IL-6 | Its increase predicts mortality from COPD (25). | High concentrations of IL- 6 increases LC risk and worsens LC prognosis (30). |
| IL-8 | Mortality was 6-times higher in patients with high WBC, CRP, IL-6, IL-8, TNF- α and fibrinogen (10). | It shows good sensitivity to diagnose LC in healthy and COPD patients. Higher concentrations increase LC risk (39). |
| Leukocytes | Mortality was 6-times higher in patients with high WBC, CRP, IL-6, IL-8, TNF- α and fibrinogen. Higher levels are associated with exacerbation risk (10). | They are increased in patients with LC+COPD when compared to COPD and healthy patients (38). |
| Lymphocytes | Lymphocyte count is significantly associated with exacerbation risk (27). | Total lymphocyte count is associated with disease progression during LC treatment (11,33). |
| Neutrophils | Neutrophils predispose towards increased inflammation and reduced bacterial clearance in COPD patients, especially during exacerbations (40). | Neutrophils are increased in patients with LC+COPD when compared to COPD and healthy patients. It is an independent indicator of poor prognosis (11,33,38). |
| NLR | NLR is higher in hospitalized non-eosinophilic COPD patients (41). | NLR is higher in LC patients when compared to healthy controls. It has been proposed as an independent prognostic factor for survival (11,33). |
| Platelets | Platelets are higher in COPD exacerbations. They have a role at predicting bacterial infections (28). | Platelets are higher in LC patients when compared to healthy controls. They are associated with poor prognosis in local and advanced LC (11). |
| MPV | MPV is higher in COPD exacerbations (42). | MPV is lower in LC patients when compared to healthy controls (11). |
| PLR | PLR is higher in COPD exacerbations. It has a role at predicting bacterial infections (28). | PLR is higher in LC patients when compared to healthy controls and it was found to be an adverse prognostic factor in NSCLC (11). |
| Fibrinogen | An increase in fibrinogen levels of 1 g/l is associated with a 3.5-fold increase in COPD mortality (10). | An elevated serum fibrinogen level was associated with poor prognosis in NSCLC (12). |
| A1AT | The congenital deficiency of alpha-1-antitrypsin is responsible for about 1% of COPD cases and 2-4% of emphysema cases (34). | Higher A1AT levels promote lung adenocarcinoma metastasis (13). |
| IgE | There is a high prevalence of elevated IgE levels in COPD patients (29). | Higher levels of IgE are associated with LC risk in healthy subjects (31). |
| CRP | Mortality was 6-times higher in patients with high WBC, CRP, IL-6, IL-8, TNF-α and fibrinogen. It increases risk of hospitalization and mortality (10). | An increase in 1 SD of the CRP levels increases the risk of dying by CP 2.32 times. Higher levels impact on survival in operable LC (32). |
| Cholesterol | Hyperlipidemia increases COPD mortality (43). | Cholesterol levels are lower in LC patients when compared to healthy controls (11) |
| Bilirubin | A reduction of 1mg/dl in bilirubin levels is associated with an increase of 86% in mortality associated with COPD (26). | Direct bilirubin levels are correlated with tumor progression, response to chemotherapy, and survival (14). |

TNF-: tumor necrosis factor; IL: interleukine; NLR: neutrophil to lymphocyte ratio; MPV: mean platelet volume; PLR: platelet to lymphocyte ratio; A1AT: alpha 1-antitripsin; IgE: E-immunoglobulin; CRP: C-reactive protein; COPD: chronic obstructive pulmonary disease; LC: lung cancer; WBC: white blood count; NSCLC: non-small cell lung cancer; SD: standard deviation.
